# To be or not to be a cytochrome: electrical characterizations are inconsistent with *Geobacter* cytochrome ‘nanowires’

**DOI:** 10.3389/fmicb.2024.1397124

**Published:** 2024-04-03

**Authors:** Matthew J. Guberman-Pfeffer

**Affiliations:** Department of Chemistry and Biochemistry, Baylor University, Waco, TX, United States

**Keywords:** *Geobacter*, nanowire, pili, cytochrome, multi-heme, OmcS, OmcZ, conductivity

## Abstract

*Geobacter sulfurreducens* profoundly shapes Earth’s biogeochemistry by discharging respiratory electrons to minerals and other microbes through filaments of a two-decades-long debated identity. Cryogenic electron microscopy has revealed filaments of redox-active cytochromes, but the same filaments have exhibited hallmarks of organic metal-like conductivity under cytochrome denaturing/inhibiting conditions. Prior structure-based calculations and kinetic analyses on multi-heme proteins are synthesized herein to propose that a minimum of ~7 cytochrome ‘nanowires’ can carry the respiratory flux of a *Geobacter* cell, which is known to express somewhat more (≥20) filaments to increase the likelihood of productive contacts. By contrast, prior electrical and spectroscopic structural characterizations are argued to be physiologically irrelevant or physically implausible for the known cytochrome filaments because of experimental artifacts and sample impurities. This perspective clarifies our mechanistic understanding of physiological metal-microbe interactions and advances synthetic biology efforts to optimize those interactions for bioremediation and energy or chemical production.

## Introduction

**Figure 1:**
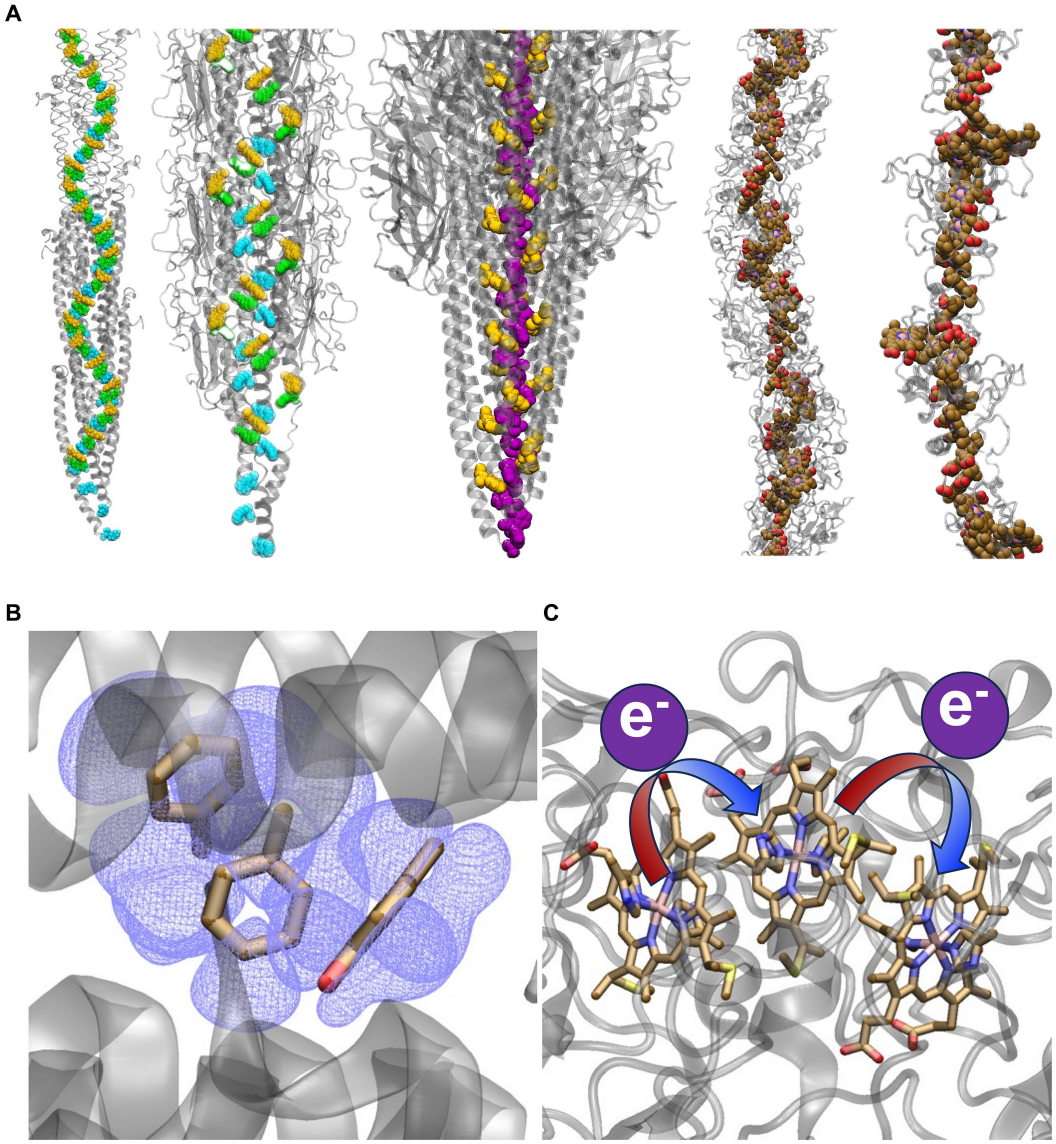
Competing structural and electrical conduction models for extracellular electron transfer. **(A)** Models of stacked Phe and Tyr residues or heme cofactors providing a conductive pathway for electrons. From left-to-right the structures are: the Arc-1 homology model of the *G. sulfurreducens* PilA-N pilus (Xiao et al., 2016) and the CryoEM resolved structure of the *G. sulfurreducens* PilA-N/PilA-C pilus (Gu et al., 2021), both with Phe-1, Phe-24, and Tyr-27 in PilA-N colored cyan, green, and yellow, respectively; CryoEM-resolved structure of the archaellum from *Methanospirillum hungatei* (Walker et al., 2019) with Phe-1 and Phe-13 colored purple and yellow respectively; and CryoEM-resolved structures of the *G. sulfurreducens* outer-membrane cytochrome types S (Wang et al., 2019) and Z (Gu et al., 2023) filaments with the hemes colored in brown. The archaellum is included to show that a continuous chain of aromatics has been observed in an experimentally-resolved structure of a natural protein assembly, which may lend some plausibility to the homology-modeled *G. sulfurreducens* PilA-N pilus. Representations of conduction via **(B)** electronic delocalization over aromatic residues or **(C)** a succession of redox reactions from one heme to the next.

Filaments from *Geobacter sulfurreducens* were reported nearly 20 years ago to be electrically conductive (Reguera et al., 2005), and yet, an intense debate persists over their identity, structure, and *in vivo* mechanism (Lovley and Walker, 2019; Reguera and Kashefi, 2019; Wang et al., 2019; Yalcin and Malvankar, 2020). Two hypotheses have divided the field (Figure 1) (Boesen and Nielsen, 2013): (1) The filament is a supramolecular assembly of cytochromes that transfers electrons through a “bucket-brigade” succession of reduction–oxidation (redox) reactions (Strycharz-Glaven et al., 2011; Boyd et al., 2015) or (2) the filament is a supramolecular assembly of PilA-N (formerly known as PilA) proteins that delocalizes electrons through a crystalloid array of stacked aromatic residues to realize metallic-like conductivity (Malvankar et al., 2011; Malvankar and Lovley, 2012).

The redox-conduction-through-cytochrome (RCTC) hypothesis is consistent with the long-established theory and function of redox cofactor chains in biology that connect catalytic centers (Page et al., 1999; Moser et al., 2000); some of these chains are even known as “molecular wire[s]” (Taylor et al., 1999; Leys et al., 2002; Pokkuluri et al., 2011). The “original hypothesis” (Tran, 2009) is supported by a wealth of spectroscopic and electrochemical data consistent with the redox chemistry of biofilms (Boyd et al., 2015), the prevalence of extracellular cytochromes expressed by *G. sulfurreducens*, and the cryogenic electron microscopy (CryoEM) determination of five filamentous cytochrome polymers from *G. sulfurreducens* (Figure 1A) (Filman et al., 2019; Wang et al., 2019, 2022a,b; Gu et al., 2023) and other prokaryotes (Baquero et al., 2023). It has also been considered implausible for crystalline-like order to be maintained among rotatable aromatic sidechains throughout a non-covalent protein assembly under ambient conditions (Strycharz-Glaven et al., 2011; Yan et al., 2015; Ru et al., 2019). The CryoEM structure of the *G. sulfurreducens* heterodimeric PilA-N/PilA-C pilus (Gu et al., 2021), in fact, shows no continuous chain of aromatic residues that can support electrical conductivity (Figure 1A).

However, heterologous expression of the *G. sulfurreducens* PilA-N protein in bacteria that do not produce outer-surface cytochromes yields (Liu et al., 2019; Ueki et al., 2020) 3-nn thinner pili with a similar diameter and conductance as 90% of the filaments emanating from *G. sulfurreducens* (Liu et al., 2021). The spectrophotometrically-detected low density of cytochromes in biofilms, as well as the immunogold-labeling and AFM-measured large spacing between (presumed) cytochrome globules on pili filaments make the RTCT hypothesis “physically impossible” (Malvankar et al., 2012b). The invariance of electrical conductivity of pili preparations to cytochrome denaturing or inhibiting conditions “definitively rule[] out” the role of *c*-type cytochromes (Malvankar et al., 2012a). A suite of experiments have shown temperature (Malvankar et al., 2011, 2014), pH (Malvankar et al., 2011, 2014; Tan et al., 2016a), voltage (Malvankar et al., 2011, 2012b), crystallinity (Malvankar et al., 2015; Xiao et al., 2016), charge propagation (Malvankar et al., 2014; Lovley and Malvankar, 2015), and aromatic density-related (Vargas et al., 2013; Adhikari et al., 2016; Tan et al., 2016a,b) dependencies of pili or biofilm conductivity—sometimes under cytochrome denaturing or inhibiting conditions—similar to synthetic organic metals (Malvankar and Lovley, 2012).

Strikingly, for example, expression of pili from other *Geobacter* spp. and point mutations in the *G. sulfurreducens* pilus caused cells to produce filaments that had a ~ 10^7^-fold range in conductivity ( $3.8\times 10^{-5}$ to $2.8\times 10^{2}$ S/cm) that correlated with aromatic residue density in the pilus (Adhikari et al., 2016; Tan et al., 2016a,b; Tan et al., 2017). For cytochromes to account for this phenomenon, a series of independent pleotropic effects would have to yield no less than five different cytochrome filaments, almost all with the same diameter (Adhikari et al., 2016; Tan et al., 2016a,b; Tan et al., 2017), that had electrical conductivities varied in precisely the way expected for the introduced pili variants.

These observations have been rationalized in terms of the metallic-like pilus (MLP) hypothesis, with the homology-modeled prediction of a seamlessly stacked array of aromatic residues (Figure 1A) (Malvankar et al., 2015; Xiao et al., 2016; Shu et al., 2017, 2019, 2020). Inspired by this picture, experiments on modified or *de novo* designed proteins lacking redox active cofactors have demonstrated, in some cases, electrical conductivities that are surprisingly high and due to mechanisms under active investigation (Creasey et al., 2015; Kalyoncu et al., 2017; Ing et al., 2018b; Shapiro et al., 2022; Krishnan et al., 2023).

But in the case of *G. sulfurreducens* biofilms and filaments, some of the experimental hallmarks of metallic-like conductivity have come to be debated on grounds of inappropriate experimental design (Bond et al., 2012; Strycharz-Glaven and Tender, 2012; Malvankar et al., 2012a, 2016; Yates et al., 2016), unreproduced by others in magnitude (Ing et al., 2017) or sign (Ing et al., 2018a), shown to depend heavily on experimental conditions (Phan et al., 2016), and found to be inconsistent with further modeling efforts (Strycharz-Glaven et al., 2011; Feliciano et al., 2012; Boesen and Nielsen, 2013; Bonanni et al., 2013; Reardon and Mueller, 2013; Lebedev et al., 2015; Yan et al., 2015; Yates et al., 2015; Ru et al., 2019). In light of the CryoEM-resolved cytochrome filament structures (Figure 1A), some (but not all) advocates of the MLP hypothesis as a “new paradigm for biological electron transfer and bioelectronics” (Malvankar and Lovley, 2012) have abandoned it in favor of the more traditional RTCT perspective.

Only a few years after reports entitled, for example, *Conductivity of Individual Geobacter Pili*, (Adhikari et al., 2016) some of the same investigators have claimed “conduction along the length of a single *bona fide* PilA filament has not been demonstrated” (Wang et al., 2019) because “[t]here has never been any direct evidence that conductive *Geobacter* extracellular filaments are composed of PilA” (Wang et al., 2019). Instead, it is now argued that filamentous outer-membrane cytochromes are “the same filaments previously thought to be TYPE IV pili” (Wang et al., 2019; Yalcin et al., 2020).

If this debated (Lovley and Walker, 2019) contention is granted, then (1) the prior cytochrome denaturation/inhibition studies that showed no change in conductivity must have been flawed, as previously charged (Strycharz-Glaven and Tender, 2012); (2) cytochromes must be capable of a 10^7^-fold variation in conductivity that coincidentally correlates with the number of aromatic residues in the *Geobacter* pilus; and (3) observations of metallic-like conductivity inconsistent with cytochromes must now either be deemed erroneous, as alleged earlier (Bond et al., 2012; Strycharz-Glaven and Tender, 2012; Yates et al., 2016), or somehow ascribed to cytochromes.

Herein, I consider if the magnitude, variation, and dependencies of the reported filament conductivities for *Geobacter* ‘nanowires’ are consistent with the CryoEM-resolved cytochrome filaments (Filman et al., 2019; Wang et al., 2019, 2022a,b; Baquero et al., 2023; Gu et al., 2023), structure-based state-of-the-art computations, (Eshel et al., 2020; Jiang et al., 2020; Livernois and Anantram, 2021; Dahl et al., 2022; Guberman-Pfeffer, 2022, 2023; Livernois and Anantram, 2023), electron transfer kinetics in multi-heme proteins (van Wonderen et al., 2019, 2021), and biological considerations (Moser et al., 2000; Page et al., 2003; Noy et al., 2006). Note that whether or not electrically conductive pili (*e*-pili) exist, and whether or not they have metallic-like conductivity are not the issues at hand. By failing to find some structural and electrical characterizations consistent with cytochromes, however, lends circumstantial support to the *e*-pilus hypothesis.

### Heme-to-heme electron transfer rates are independent of filament identity

Thousands of successive redox reactions are thought to move electrons through micron-scale filamentous cytochromes under physiological conditions (Blumberger, 2018). In each reaction, electrons ‘hop’ between weakly (<0.02 eV) coupled hemes (Blumberger, 2018; Jiang et al., 2020; Dahl et al., 2022; Guberman-Pfeffer, 2022; Guberman-Pfeffer, 2023) packed in highly conserved T- and slip-stacked geometries (Supplementary Tables S1, S2; Supplementary Figure S1) (Baquero et al., 2023). The van der Waals-packing permits the surrounding protein/water media to impose only a small (<|0.3| eV) free energy difference on the heme-to-heme self-exchange reaction (Supplementary Tables S3, S4) (O'Brien, 2020; Dahl et al., 2022; Shipps, 2022; Guberman-Pfeffer, 2023). A > 0.4 eV cost is also imposed for the reorganization of the environment to the altered charge distribution in the reaction (Supplementary Table S5) (Jiang et al., 2020; Dahl et al., 2022; Guberman-Pfeffer, 2023). The lowering of this penalty by active site polarizability is found to be minimal (~0.04 eV; Supplementary Tables S6–S8). This picture is derived from spectroelectrochemical experiments (O'Brien, 2020; Shipps, 2022) and structure-based calculations (Jiang et al., 2020; Dahl et al., 2022; Guberman-Pfeffer, 2022; Guberman-Pfeffer, 2023) on the CryoEM-resolved cytochrome filaments.

**Figure 2:**
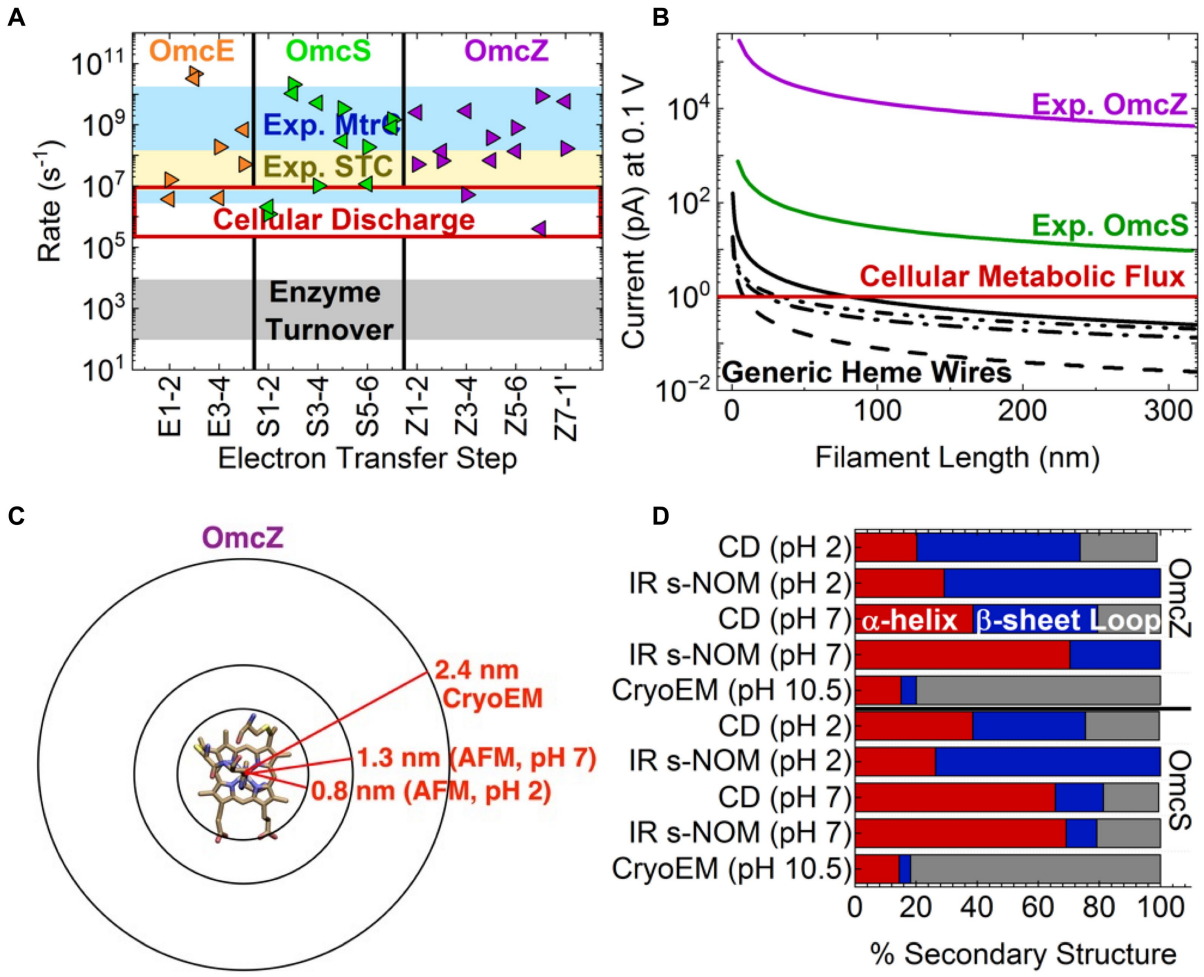
Inconsistencies in the electrical and structural characterizations of cytochrome filaments. *Top*: Heme-to-heme electron transfer rates are independent of protein identity and meet or exceed cellular metabolic flux but are cannot account for than reported conductivities. **(A)** Comparison of computed structure-based Marcus theory rates for the outer-membrane cytochrome (Omc) types E, S, and Z from *Geobacter sulfurreducens* (Guberman-Pfeffer, 2023) to experimentally measured rates for the same highly conserved heme packing geometries in the small tetraheme cytochrome (STC; van Wonderen et al., 2019) and metal reducing cytochrome type C (MtrC; van Wonderen et al., 2021) from *Shewanella oneidensis*. **(B)** The length dependence of the protein-limited current at 0.1 V through generic heme wires compared to the currents through Omc- S and Z based on the reported conductivities (Yalcin et al., 2020; Dahl et al., 2022). The solid, dashed, dashed-dot, and dashed-dot-dot curves, respectively, correspond to heme chains with all slip-stacked, all T-stacked, strictly alternating slipped and T-stacked, and a T → S → S → T → S → T → S (T = T-stacked; S = slip-stacked) pattern as found in OmcZ. Computational details accompany Supplementary Table S11. *Bottom*: Infrared nanospectroscopy using scattering-type scanning near-field optical microscopy (IR s-NOM), circular dichroism (CD) and atomic force microscopy (AFM) structural characterizations are inconsistent with the cryogenic electron microscopy (CryoEM)-resolved filaments. **(C)** Comparison of filament radii for OmcZ measured by CryoEM and AFM at different pHs. **(D)** Secondary structure compositions reported by CryoEM, IR s-NOM, and CD at different pHs. The data is reproduced from Yalcin et al. (2020).

The energetic constraints ensure the applicability of non-adiabatic Marcus theory (Blumberger, 2018) and encode ground-state inter-heme electron transfers on the hundreds of ns to 𝜇s timescales (Figure 2; Supplementary Table S9). For all computations to date (Jiang et al., 2020; Dahl et al., 2022; Guberman-Pfeffer, 2023) reaction rates between T- and slip-stacked heme pairs in the filaments are on average $5.3\times 10^{7}$ to $2.0\times 10^{9}$ s^−1^ and $3.0\times 10^{9}$ to $2.0\times 10^{10}$ s^−1^, respectively (Supplementary Table S10). These rates are in excellent agreement with the average rates derived from kinetic analyses of ultrafast transient absorption measurements on photosensitized variants of the small tetraheme cytochrome (STC) (van Wonderen et al., 2019) and the metal reducing cytochrome type C (MtrC) (van Wonderen et al., 2021) from *Shewanella oneidensis*. As anticipated by Blumberger and co-workers (van Wonderen et al., 2021) and Page, Moser, and Dutton two decades earlier (Page et al., 1999), inter-heme electron transfer rates within highly conserved packing geometries are also highly conserved.

### Evolution favors robustness over tunability

Independence from the surrounding protein affords evolutionary robustness at the expense of tunability, which seems tolerable because the rates already exceed typical timescales for enzymatic turnover (≥ 𝜇s vs. ms; Figure 2A) (Noy et al., 2006), and do not therefore pose the rate-limiting step for cellular respiration (Page et al., 2003). Indeed, if the cytochrome filaments are optimized for electrical conductivity, why do nearly half (Filman et al., 2019; Wang et al., 2019, 2022a,b; Baquero et al., 2023; Gu et al., 2023) of all heme pairs adopt a T-stacked geometry that enforces 10-fold slower electron transfers than a slip-stacked geometry? It is important not to fall into the Panglossian paradigm (Page et al., 2003).

Functional robustness permits adaptation of the environment-filament interface formed by the surrounding protein without having to re-invent the mechanism for long-range electron transfer. This “design”-strategy is analogous to how the conserved photosystems of photosynthesis are interfaced to distinct spectral niches by highly adapted light harvesting antenna (Guberman-Pfeffer, 2023). Finding modular solutions to decoupled problems is a meta-strategy of evolution. An implication is that *de novo* design seems to be a more promising avenue for application-tailored cytochrome ‘nanowires’ than mutagenesis.

### A minimum of seven cytochrome filaments/cell can carry the total metabolic current

Theory and experiment both suggest that $1\times 10^{8}$ and $1\times 10^{9}$ s^−1^ are protein-independent, order-of-magnitude estimates for ground-state heme-to-heme electron transfer in T- and slip-stacked geometries, respectively. Using these generic rates, the experimentally characterized 300 nm-long filaments are predicted to support protein-limited currents of ~0.14 pA (Figure 2B; Supplementary Table S11), a result somewhat underestimated by structure-based calculations (Supplementary Figure S2). A *G. sulfurreducens* cell discharging ~1 pA (i.e., oxidizing ~ $8\times 10^{5}$ acetate molecules/s) would require a minimum of ~7 cytochrome filaments; somewhat more (≥20 filaments/cell) are likely expressed to increase the chance of productive contacts with (microbial or mineral) electron sinks (Reguera et al., 2005; Summers et al., 2010; Reguera and Kashefi, 2019).

### Chemical, not electrical gradients drive electrons through cytochrome filaments

An open question with the conventional picture of redox conduction is what provides the needed driving force for a succession of thousands of reactions to transfer electrons on the micrometer scale. Each one-electron heme-to-heme step incurs a reorganization penalty at least quadruple the ~0.1 V drop between the intra-cellular acetate oxidation and extracellular iron oxide reduction half reactions connected by the filament. This penalty accumulates along the filament length, while heme-to-heme reaction free energies are nearly net-zero through a filament subunit (Supplementary Table S4). The external bias is energetically smaller than thermal noise between any two adjacent hemes (if linearly interpolated along the filament) and furthermore screened by mobile ions. The exodus of $3\times 10^{5}$ – $9\times 10^{6}$ electrons/s/cell (Lampa-Pastirk et al., 2016; Karamash et al., 2022) must, it seems, provide the needed driving force in the form of a concentration gradient.

### Current electrical characterizations reflect abiological artifacts

But heme-Fe redox activity (Amdursky et al., 2013; Blumberger, 2018; Agam et al., 2020; Futera et al., 2020) and concentration gradients (Strycharz-Glaven et al., 2011; Bostick et al., 2018) are not relevant under the electron transport (as opposed to transfer) conditions of the performed conducting probe atomic force microsocpy (CP-AFM) experiments; namely electrode adsorbed, air-dried, and mechanically compressed filaments exposed to electric fields unscreened by mobile ions. Some other mechanism may be operative. In support of this hypothesis, the measured currents are 10^2^–10^5^-fold larger than the computed and biologically reasonable maximum redox current of 0.14 pA/filament (Figure 2B), even at a voltage well-below the protein-limited threshold (Wang et al., 2019; Yalcin et al., 2020; Dahl et al., 2022). Contrary to some (Dahl et al., 2022) but not all (Eshel et al., 2020; Guberman-Pfeffer, 2022; Guberman-Pfeffer, 2023) prior claims, a physiologically relevant succession of redox reactions (multi-step hopping) cannot even come close to account for the reported conductivities.

Moreover, the 30 nA current reported at 0.1 V for OmcZ (Yalcin et al., 2020) requires an effective electron transfer rate of $3\times 10^{12}$ s^−1^. This rate is nearly at the non-adiabatic electronic coupling-maximum ‘speed limit’ of 10^13^ s^−1^ for metal ions in van der Waals contact (Gray and Winkler, 2010), even though the Fe centers are separated by at least triple that distance. Only if active-site polarizability reduces the computed reorganization energies by 45% (Kontkanen et al., 2023), every heme-to-heme electron transfer is activationless, and all electronic couplings are 20-fold larger than computed from the CryoEM structure can the measured conductivity be explained by the redox process. There is no basis for the hypothesis that increased electronic couplings due to some hemes being ~1.0 Å closer can account for the 10^3^-fold greater conductivity of Omc- Z versus S (Guberman-Pfeffer, 2023).

Of note, there was also no basis for the reported conductivities when Omc- Z and S were argued to be the F51W-F57W and wild-type pili, respectively (Tan et al., 2016a). An increase in aromatic density was then claimed to explain the higher conductivity of the F51W-F57W pilus by erroneously counting the substitution of one aromatic residue (Trp) for a different aromatic residue (Phe) as introducing an additional aromatic residue.

In addition to the abiological electron transport conditions used experimentally, the reported conductivities may be artificially large because the 40–60 nm-wide tip used in CP-AFM can contact ~10 filament subunits, or ~ 10^2^ hemes along its diameter. The observed current scales linearly with the number of electrode-protein contacts (Polizzi et al., 2012). Also, a force of 10–50 nN was applied to the filaments in CP-AFM experiments (Yalcin et al., 2020; Dahl et al., 2022), which is known to mechanically deform much more structured proteins (e.g., azurin, plastocyanin, and cytochrome *c*) (Zhao and Davis, 2003; Zhao et al., 2004; Andolfi and Cannistraro, 2005; Bonanni et al., 2005; Axford et al., 2007; Li et al., 2012), and change the electron transport mechanism; for example, by changing the packing density. More force can increase the packing density, which in turn promotes transmission of electrons. In this context, the experimental procedure of measuring the length dependence of the resistance by passing the current through previously crushed segments of the filament (Yalcin et al., 2020; Dahl et al., 2022) is problematical. The cytochrome filaments are potentially more responsive to compressional force than previously studied proteins because ≥50% of the secondary structure consists of flexible turns and loops (Filman et al., 2019; Wang et al., 2019, 2022a,b; Baquero et al., 2023; Gu et al., 2023).

### Cytochromes cannot logically have properties inconsistent with cytochromes

Several other characterizations of *Geobacter* ‘nanowires’ may also be experimental artifacts if the contention is granted that the filaments have always been cytochromes.

#### Carbon nanotube-like charge delocalization supported by crystalline π-π stacking

Electrostatic force microscopy (EFM) experiments showed holes delocalize rapidly for microns along individual filaments and accumulate in what were described as cytochrome-like globules on the filament surface (Malvankar et al., 2014). Extensive delocalization was attributed to the crystalline order of π-π stacking interactions that control the conjugation length or mean free path of electrons (Malvankar et al., 2011, 2015; Yalcin et al., 2020).

Electrons in cytochromes, however, propagate through spatially confined and weakly coupled heme-centered molecular orbitals that participate in discrete redox transitions under physiological conditions. The reported conductivities are much too small to justify a band-theory electronic description (Polizzi et al., 2012). Instead, the EFM results may be an artifact of the ±10 V bias used to inject charges into the filaments (Malvankar et al., 2014),which accessed electronic excited states completely forbidden to biology.

#### Synthetic metal-like voltage dependent conductivity

Electrochemical gating showed an organic metal-like sigmoidal voltage dependence with no peak in conductivity at the formal potential and an increase at highly oxidizing potentials (Malvankar et al., 2012b). Cytochromes, as redox conductors, have a strongly peaked conductivity at the formal potential that falls to zero at the extremes where populations of both charge donating and accepting hemes are unavailable (Strycharz-Glaven et al., 2011). By contrast, highly oxidizing potentials were suggested to be necessary for conductivity in the homology-modeled *G. sulfurreducens* pilus (Ru et al., 2019).

More recently, a 6-fold higher conductivity of fully reduced versus fully oxidized OmcS was rationalized by invoking a 0.3–0.6 V electrochemical hysteresis for each heme (Supplementary Table S3) (Dahl et al., 2022). This hysteresis was not observed in two independent sets of spectroelectrochemical experiments (O'Brien, 2020; Shipps, 2022) performed in the investigators’ laboratory years before publishing this explanation. Anantram and co-workers (Livernois and Anantram, 2023) have instead proposed that the redox-state-dependent conductivity is because of improved alignment and coupling of the heme orbitals with the Fermi level of the electrodes upon reduction, an effect with no biological relevance.

#### Synthetic metal-like temperature dependent conductivity

The conductivity of biofilms (Malvankar et al., 2011), films of filaments (Malvankar et al., 2011), and individual filaments (Dahl et al., 2022) exponentially increased upon cooling down to a cross-over temperature, below which the conductivity exponentially decreased upon further cooling. This behavior, which has not been reproduced by others (Ing et al., 2018a) and may strongly depend on uncontrolled humidity in the experiments (Phan et al., 2016) was ascribed to delocalized electrons that move freely without thermal activation and experience reduced phonon scattering upon cooling, but encounter disorder, or localizing traps at temperatures below the crossover point, as in synthetic organic metals (Kaiser, 2001).

Redox conduction in cytochromes, by contrast, is expected to show Arrhenius-type kinetics, as computationally demonstrated by the present author for the OmcS filament (Guberman-Pfeffer, 2022). Other computational work (Dahl et al., 2022) found that if unphysically negative redox potentials in contradiction to spectroelectrochemical analyses (O'Brien, 2020; Shipps, 2022) are considered, the conductivity at 310 versus 270 K can be severely underestimated, and thereby give the impression of validating the experimental temperature dependence.

#### Increased conductivity at cytochrome denaturing pHs

Filaments from *G. sulfurreducens* became more conductive upon acidification from pH 7 to 2 (Malvankar et al., 2011; Tan et al., 2016a). Redox conduction in cytochromes may become more favorable with an increased H^+^ concentration if the electron and proton transfers are coupled. The reported kinetic isotope effect (KIE) on conductivity in OmcS was similar to other proton-coupled-electron-transfer (PCET) systems, and the present author found 26–42% charge compensation from coupled protonation and redox state changes (Guberman-Pfeffer, 2022). Dynamical solvent control may also contribute to the KIE (Mostajabi Sarhangi and Matyushov, 2023).

Cytochromes, however, are expected to denature at pH 2. Furthermore, circular dichroism (CD) spectra of OmcS in pH 7 potassium phosphate and pH 2 sodium citrate buffers were more similar to one another than to CD spectra under solid-state conditions (Yalcin et al., 2020). The pH-dependence, which is already physiologically irrelevant at pH 2, may be an artifact of surface adsorption and dehydration.

These artifacts may manifest as the observed structural changes (Figures 2C,D); namely (1) a ~ 1.0 nm shrinkage in filament diameter from pH 7 to 2 that left OmcZ no wider than a single heme group, as if the protein had been hydrolyzed away, and (2) an 𝛼-helical-to- β -sheet transition quantified with techniques that disagreed on the magnitude of the effect (50% versus ≤21% conversion) (Yalcin et al., 2020). How, and energetically why such a large structural change of the polypeptide backbone can be accommodated by the geometrical constraints of covalent thioether linkages and coordinative His-Fe bonds to each heme is difficult to comprehend.

### Electrical measurements have not been performed on known cytochrome filament structures

Some unknown and abiological mechanism in cytochromes may be operative under experimental conditions to account for these observations. However, it is also true that spectroscopic characterizations of protein structure in the same study (Yalcin et al., 2020) that reported electrical measurements disagree with the CryoEM analyses (Figure 2B) (Filman et al., 2019; Wang et al., 2019, 2022a,b; Baquero et al., 2023; Gu et al., 2023). Infrared nanospectroscopy using scattering-type scanning near-field optical microscopy (IR s-SNOM) and CD measurements on purportedly OmcS filaments indicated 66–69% (instead of 14%) α -helical content, 10–16% (instead of 4%) β -sheet content, and 18–21% (instead of 82%) loops/turns. For purportedly OmcZ filaments, α -helical content was 70% by IR s-NOM and 39% by CD in the same study, instead of 15% by CryoEM; β -sheet content was 30% by IR-SNOM and 41% by CD, instead of 5% by CryoEM; and neither IR s-NOM nor CD found any of the 80% loops/turns witnessed by CryoEM.

The discrepancies of IR s-NOM (but not CD) were attributed to a particular sensitivity of the technique to C=O versus N-H stretching (Yalcin et al., 2020), but this explanation is both technically inaccurate and inapplicable. IR s-NOM “primarily probes molecular vibrations that oscillate perpendicular to the sample surface” (Amenabar et al., 2013), which only translates to a particular sensitivity to C=O versus N-H stretching in the case of an oriented protein, such as membrane-embedded bacteriorhodopsin in the original publication for the technique. The orientation of the putative cytochrome filaments, by contrast, was not controlled for the experiments in any way.

It was furthermore claimed that secondary structure percentages from IR s-NOM are only quantitative in a comparative sense because of the alleged particular sensitivity of the technique (Yalcin et al., 2020). If true, why did IR s-NOM and CD find 19 and 10% more β -sheet content in Omc- Z versus S, respectively, whereas CryoEM found the same amount of β -sheet content in the two proteins (Wang et al., 2022a; Gu et al., 2023)? Of note, a false impression was given that there is no discrepancy by stating (Yalcin et al., 2020)—at odds with the accompanying structure (PDB 7LQ5)—that the CryoEM model of OmcZ has 21 instead of 5% β -strands and by wrongly counting β -turns as regular secondary structure.

## Discussion

The structural discrepancies relative to CryoEM, on top of the physiological irrelevance or physical implausibility of electrical characterizations for a multi-heme architecture beg of the filaments the title question: To be or not to be a cytochrome?

The foregoing evidence suggests answering in the affirmative: A handful of cytochrome filaments resolved by CryoEM can carry the entire metabolic flux of electrons from a *Geobacter* cell at rates that are both consistent with kinetic analyses on other multi-heme proteins and do not pose the rate-limiting step to cellular respiration. Cytochrome filaments are proposed to be physiologically relevant. Meanwhile, the reported 10^2^–10^5^-fold larger conductivities, the 10^7^-fold variation in conductivity with PilA-N aromatic density, the hallmarks of metallic-like charge propagation, voltage, temperature, and pH dependences, and the spectroscopically-deduced secondary structure compositions are all irreconcilable with the known cytochrome filaments.

The conclusion follows that earlier claims of inappropriate experimental design are true (Boyd et al., 2015), biologically irrelevant phenomena have been measured, and/or the conformation or composition of the characterized proteins is something other than the known cytochrome filaments. Experiments designed to assess the redox-based electrical conductivity of well-characterized (e.g., composition, purity, structure) filament samples under physiologically relevant conditions are urgently needed. Just as urgently needed are efforts to independently reproduce the electrical characterizations that have been the claims of only a few laboratories.

## Data availability statement

The original contributions presented in the study are included in the article/Supplementary material, further inquiries can be directed to the corresponding author.

## Author contributions

MJG-P: Conceptualization, Data curation, Formal analysis, Investigation, Methodology, Visualization, Writing – original draft, Writing – review & editing.
