# Supplementary material for: To be or not to be a cytochrome: electrical characterizations are inconsistent with Geobacter cytochrome ‘nanowires’
Source: Front Microbiol. 2024 Apr 3;15:1397124. doi: 10.3389/fmicb.2024.1397124 (PMC11021709; doi:10.3389/fmicb.2024.1397124)
Supplement: Supplementary file 1 [file Data_Sheet_1.pdf]

Supporting Information

To be or Not to be a Cytochrome: Electrical Characterizations are Inconsistent with *Geobacter* cytochrome 'nanowires'?

Matthew J. Guberman-Pfeffer[a,]*

[a]Department of Chemistry and Biochemistry, Baylor University, One Bear Place #97348, Waco, Texas 76706, United States;

**** Author to whom correspondence should be addressed.***

E-mail: Matthew_Guberman-Pfe@baylor.edu

KEYWORDS: Geobacter, nanowire, pili, cytochrome, multi-heme, OmcS, OmcZ, conductivity

Table 1. Metrical Parameters for the heme centers in Omc- E, S, and Z from classical molecular dynamics simulations reported in Ref. 1. All the quantities are consistent with the highly conserved heme stacking geometries identified through a survey of >800 cytochromes in Ref. 2. Note that a double prime mark is used to indicate the heme in the next subunit of the filament.

Heme Pair	Packing Designation	Edge-to-Edge Distance (Å)	Fe-to-Fe Distance (Å)	Heme Rotation (°)	Heme Plane Tilt (°)	His-His Rotation (°)
OmcE						
1↔2	T	6.1 ± 0.3	11.8 ± 0.4	136.3 ± 3.1	73.4 ± 4.5	32.1 ± 22.2, 16.2 ± 9.9
2↔3	S	3.7 ± 0.2	9.1 ± 0.3	178.0 ± 1.5	18.7 ± 4.4	16.2 ± 9.9, 20.0 ± 10.0
3↔4	T	6.0 ± 0.2	11.7 ± 0.4	145.5 ± 2.3	86.0 ± 3.2	20.0 ± 10.0, 72.7 ± 9.6
4↔1’’	S	4.4 ± 0.3	10.0 ± 3.3	172.0 ± 2.8	13.8 ± 4.9	72.7 ± 9.6, 23.1 ± 11.9
OmcS						
1↔2	T	6.0 ± 0.2	12.6 ± 0.2	130.7 ± 3.0	69.1 ± 2.6	12.0 ± 9.1, 28.3 ± 11.2
2↔3	S	3.9 ± 0.2	9.4 ± 0.2	176.7 ± 1.7	11.0 ± 2.0	28.3 ± 11.2, 63.5 ± 10.1
3↔4	T	6.0 ± 0.2	11.3 ± 0.2	143.4 ± 2.2	75.2 ± 2.3	63.5 ± 10.1, 42.5 ± 11.2
4↔5	S	3.9 ± 0.2	9.5 ± 0.2	176.7 ± 1.8	8.0 ± 2.9	42.4 ± 11.2, 23,9 ± 12.0
5↔6	T	5.9 ± 0.2	11.3 ± 0.2	142.2 ± 2.3	82.2 ± 2.7	23.9 ± 12.0, 66.4 ± 10.9
6↔1'’	S	4.0 ± 0.2	9.7 ± 0.2	177.8 ± 1.6	7.7 ± 2.6	66.4 ± 10.9, 13.6 ± 8.2
OmcZ						
1↔2	T	5.5 ± 0.1	11.4 ± 0.2	137.7 ± 3.3	68.8 ± 2.5	16.8 ± 8.9, 72.3 ± 10.2
2↔3	S	4.0 ± 0.2	9.8 ± 0.2	172.9 2.1	11.8 ± 2.4	72.3 ± 10.2, 45.9 ± 15.3
3↔8	B	5.3 ± 0.3	10.4 ± 0.3	96.5 ± 3.9	51.0 ± 3.5	45.9 ± 15.3, 40.9 ± 19.7
3↔4	S	4.2 ± 0.2	10.3 ± 0.2	56.5 ± 2.6	26.0 ± 3.1	45.9 ± 15.3, 12.5 ± 6.6
4↔5	T	4.9 ± 0.2	10.0 ± 0.3	172.4 ± 4.1	75.9 ± 4.2	12.5 ± 6.6, 16.4 ± 9.4
5↔6	S	4.0 ± 0.3	9.9 ± 0.4	160.8 ± 3.5	19.1 ± 3.9	16.4 ± 9.4, 57.2 ± 11.8
6↔7	T	5.3 ± 0.3	11.4 0.3	121.2 ± 4.0	75.4 ± 3.5	57.2 ± 11.8, 41.0 ± 17.9
7↔1'’	S	3.9 ± 0.2	9.4 ± 0.2	170.0 ± 2.4	23.3 ± 3.1	41.0 ± 17.9, 13,1 ± 7.2

Table S2. Electronic couplings computed for Omc- E, S, And Z surveyed from the literature.[1, 3, 4] The present author's name (Guberman-Pfeffer) is abbreviated as G-P for conciseness.

Author	Heme-to-Heme Electronic Coupling (meV)								
					OmcE				
Heme Pair	4'↔1	1↔2	2↔3	3↔4	4↔1''				
G-P.	7.80	1.44	12.85	1.87	4.41				
					OmcS				
Heme Pair	6'↔1	1↔2	2↔3	3↔4	4↔5	5↔6	6↔1'		
G-P	6.72	1.13	6.09	2.64	9.52	1.04	7.90		
Dahl et al.		8.93	15.9	3.48	12.4	5.68	8.76		
Jiang et al.	3.94	1.54	9.35	2.12	6.07	1.18			
					OmcZ				
Heme Pair	7'↔1	1↔2	2↔3	3↔4	4↔5	5↔6	6↔7	7↔1''	3↔8
G-P.	9.83	2.04	3.19	7.15	4.06	5.21	2.32	4.71	2.43

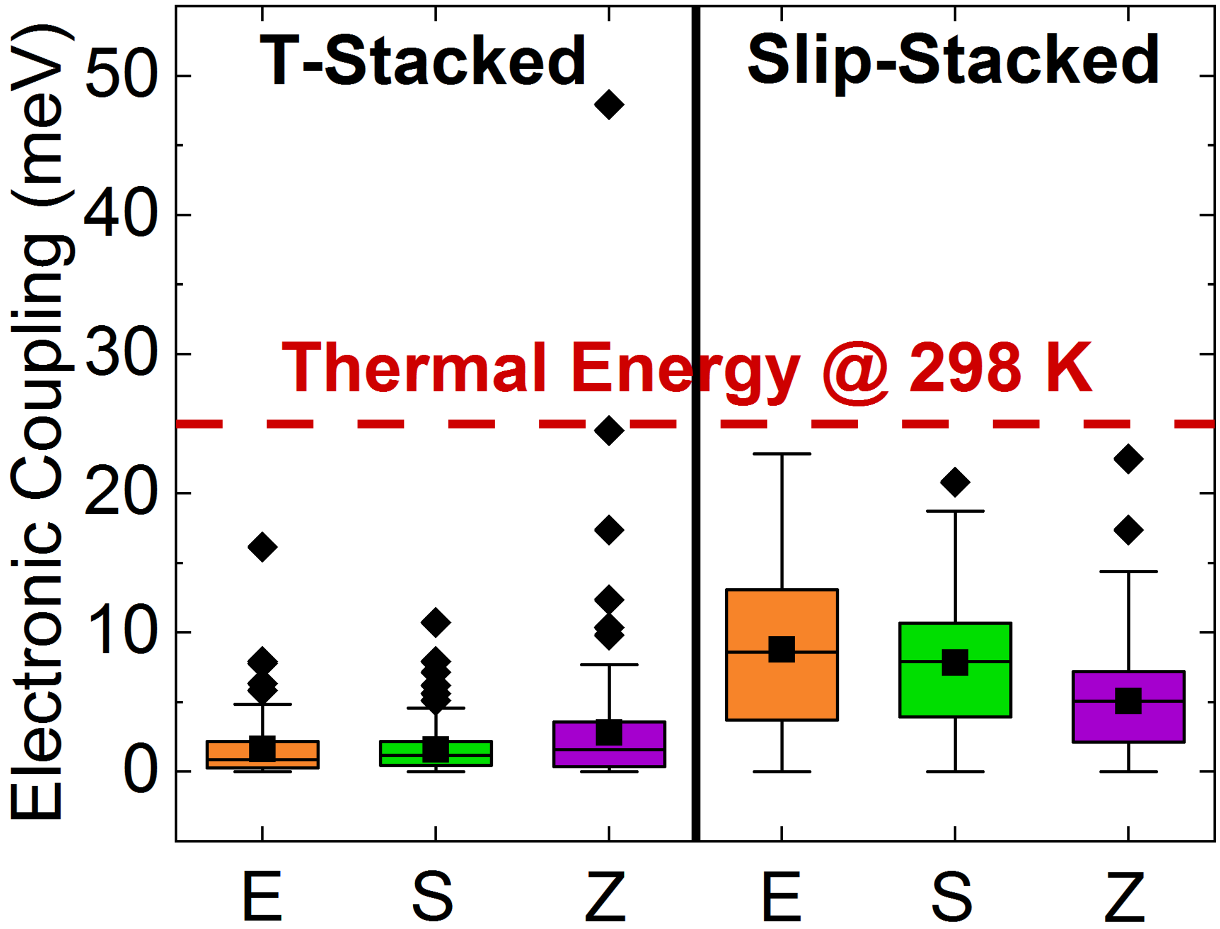

Figure S1. Distributions of computed electronic couplings for Omc- E, S, and Z according to the packing geometry (T- or slip-stacked) of the donor-acceptor heme pair. The data is reproduced from Ref.1 .

Table S3. Comparison of spectroelectrochemically-derived and computed redox potentials. All values are in V vs. SHE. The parenthetical numbers indicate the heme to which the computed potential belongs. The spectroelectrochemical data is reproduced from Ref. [5]. The computed potentials from the present author (Guberman-Pfeffer) are reproduced from Ref. [6]; those from Dahl *et al.* are reproduced from Ref. 4. The difference in potential between adjacent hemes determines the free energy, and therefore, this comparison to experiment is important for assessing the computed ΔGs in Table X.

		Cathodic Direction		Anodic Direction
		Guberman-Pfeffer	Dahl *et al.*	Dahl et al.
Site	Experiment	QM/MM@MD	QM/MM@MD	QM/MM@MD
1	-0.070	-0.108 ± 0.023 (#3)	-0.080 ± 0.06 (#6)	-0.421 ± 0.05 (#2)
2	-0.121	-0.119 ± 0.027 (#1)	-0.108 ± 0.04 (#2)	-0.431 ± 0.06 (#6)
3	-0.138	-0.128 ± 0.028 (#6)	-0.126 ± 0.05 (#3)	-0.491 ± 0.05 (#1)
4	-0.168	-0.130 ± 0.023 (#2)	-0.191 ± 0.05 (#1)	-0.647 ± 0.05 (#4)
5	-0.220	-0.214 ± 0.025 (#5)	-0.334 ± 0.04 (#4)	-0.727 ± 0.05 (#3)
6	-0.281	-0.271 ± 0.027 (#4)	-0.521 ± 0.05 (#5)	-0.826 ± 0.05 (#5)
E°_{app}	-0.155	-0.146	-0.166	-0.570

[a]The present study is chiefly concerned with the cathodic direction because most conductivity experiments are performed in the air-oxidized state. The computed anodic currents from Dahl *et al.* are also shown, however, because they too conflict with the spectroelectrochemical data that found a minimal (tens of mV at most) hysteresis.

Table S4. Reaction free energies (eV) computed for Omc- E, S, and Z surveyed from the literature.[1, 3, 4] The present author's name (Guberman-Pfeffer) is abbreviated G-P for conciseness. G-P and Dahl *et al.* computed the reaction free energies using vertical energy gaps from quantum mechanical/molecular mechanical calculations at classical molecular dynamics-generated configuration (QM/MM2MD). However, as noted before (Ref [6]), the level of QM model chemistry, the number of evaluated configurations, and other technical aspects had important differences between the two studies. Jiang *et al.* approximated the reaction free energy as the redox-linked difference in electrostatic energy from Poisson-Boltzmann calculations.

Author	Heme-to-Heme Electronic Coupling (meV)								
					OmcE				
Heme Pair	4'↔1	1↔2	2↔3	3↔4	4↔1''				
G-P.	0.188	-0.038	-0.009	-0.099	0.067				
					OmcS				
Heme Pair	6'↔1	1↔2	2↔3	3↔4	4↔5	5↔6	6↔1'		
G-P	0.031	0.014	-0.017	0.161	-0.063	-0.072	-0.015		
Dahl et al.		0.018	0.209	0.186	-0.440	0.111			
Jiang et al.	0.120	-0.090	0.000	0.090	-0.040	-0.090			
					OmcZ				
Heme Pair	7'↔1	1↔2	2↔3	3↔4	4↔5	5↔6	6↔7	7↔1"	3↔8
G-P.	0.189	0.101	0.019	0.163	-0.044	-0.046	-0.258	0.092	

Table S5. Reorganization energies (eV) computed for Omc- E, S, and Z surveyed from the literature.[1, 3, 4] The present author's name (Guberman-Pfeffer) is abbreviated as G-P for conciseness. G-P and Dahl *et al.* computed the reorganization energies using vertical energy gaps computed along classical molecular dynamics trajectories. Jiang *et al.* used an approximation to the Marcus continuum expression that parameterizes the static dielectric constant in terms of a linear dependence on the solvent accessible surface area, where the parameters were developed so that the reorganization energies reproduce those obtained from polarizable classical molecular dynamics.

Author	Heme-to-Heme Electronic Coupling (meV)								
				OmcE					
Heme Pair	4'↔1	1↔2	2↔3	3↔4	4↔1''				
G-P.	0.688	0.879	0.481	0.780	0.778				
				OmcS					
Heme Pair	6'↔1	1↔2	2↔3	3↔4	4↔5	5↔6	6↔1'		
G-P	0.668	0.983	0.429	0.626	0.765	0.630	0.724		
Dahl et al.		0.605	0.578	0.658	0.829	0.819	0.675		
Jiang et al.	0.66	0.86	0.64	0.71	0.60	0.78			
				OmcZ					
Heme Pair	7'↔1	1↔2	2↔3	3↔4	4↔5	5↔6	6↔7	7↔1"	3↔8
G-P.	0.593	0.556	0.785	0.892	0.781	0.758	0.680	0.624	

Table S6. Vertical energy gaps and reorganization energies (in eV) for the hemes of OmcE with self-consistently optimized electron densities in vacuum and the protein environment, or with the density frozen at the vacuum-optimized distribution while the heme is in the protein context. The data is reproduced from Ref. 1.

Heme	Relaxed Density in Vacuum			Frozen Vacuum density In Protein Environment			Relaxed Density in Protein environment		
#	-VEA	VIP	λ	-VEA	VIP	λ	-VEA	VIP	λ
4’	4.881 ± 0.008	4.928 ± 0.009	0.023	3.250 ± 0.019	4.878 ± 0.018	0.814	3.239 ± 0.018	4.889 ± 0.019	0.825
1	4.874 ± 0.008	4.852 ± 0.008	-0.011	2.961 ± 0.018	4.742 ± 0.020	0.891	3.013 ± 0.019	4.739 ± 0.020	0.863
2	4.869 ± 0.007	4.917± 0.009	0.024	3.008 ± 0.021	4.763 ± 0.019	0.878	3.064 ± 0.023	4.763 ± 0.020	0.850
3	4.808 ± 0.006	4.816 ± 0.009	0.004	3.084 ± 0.019	4.705 ± 0.021	0.811	3.135 ± 0.021	4.710 ± 0.020	0.788
4	4.860 ± 0.007	4.944 ± 0.009	0.042	3.168 ± 0.021	4.836 ± 0.040	0.834	3.181 ± 0.021	4.862 ± 0.018	0.841
1’’	4.882 ± 0.008	4.863 ±0.008	-0.009	2.970 ± 0.024	4.871 ± 0.023	0.951	3.044 ± 0.024	4.865 ± 0.021	0.911

Table S7. Vertical energy gaps and reorganization energies (in eV) for the hemes of OmcS with self-consistently optimized electron densities in vacuum and the protein environment, or with the density frozen at the vacuum-optimized distribution while the heme is in the protein context. The data is reproduced from Ref. 1.

Heme #	Relaxed Density in Vacuum			Frozen Vacuum density In Protein Environment			Relaxed Density in Protein environment		
	-VEA	VIP	λ	-VEA	VIP	λ	-VEA	VIP	λ
6’	4.949 ± 0.008	4.930 ± 0.007	-0.010	3.449 ± 0.019	5.002 ± 0.017	0.777	3.464 ± 0.020	5.003 ± 0.016	0.770
1	4.888 ± 0.007	4.904 ± 0.008	0.008	3.326 ± 0.021	5.027 ± 0.019	0.851	3.372 ± 0.021	5.033 ± 0.020	0.831
2	4.900 ± 0.007	4.912 ± 0.008	0.006	3.327 ± 0.016	5.062 ± 0.018	0.868	3.329 ± 0.016	5.047 ± 0.017	0.859
3	4.834 ± 0.006	4.856 ± 0.006	0.011	3.530 ± 0.015	4.825 ± 0.016	0.648	3.555 ± 0.016	4.856 ± 0.015	0.651
4	4.943 ± 0.008	4.932 ± 0.008	-0.005	3.344 ± 0.018	4.692 ± 0.018	0.674	3.398 ± 0.019	4.691 ± 0.018	0.647
5	4.870 ± 0.007	4.829 ± 0.007	-0.021	3.342 ± 0.019	4.889 ± 0.018	0.774	3.333 ± 0.018	4.881 ± 0.018	0.774
6	4.932 ± 0.008	4.935 ± 0.009	0.001	3.392 ± 0.019	4.922 ± 0.018	0.765	3.421 ± 0.019	4.938 ± 0.019	0.759
1’’	4.869 ± 0.007	4.888 ± 0.007	0.010	3.334 ± 0.019	5.027 ± 0.018	0.847	3.356 ± 0.020	5.032 ± 0.018	0.838

Table S8. Vertical energy gaps and reorganization energies (in eV) for the hemes of OmcZ with self-consistently optimized electron densities in vacuum and the protein environment, or with the density frozen at the vacuum-optimized distribution while the heme is in the protein context. The data is reproduced from Ref. 1.

Heme #	Relaxed Density in Vacuum			Frozen Vacuum density In Protein Environment			Relaxed Density in Protein environment		
	-VEA	VIP	λ	-VEA	VIP	λ	-VEA	VIP	λ
7'	4.869 ± 0.007	4.879 ± 0.007	0.005	3.148 ± 0.021	5.126 ± 0.018	0.989	3.207 ± 0.022	5.146 ± 0.018	0.970
1	4.884 ± 0.008	4.877 ± 0.006	-0.004	3.147 ± 0.022	4.797 ± 0.016	0.825	3.170 ± 0.024	4.806 ± 0.016	0.818
2	4.848 ± 0.007	4.862 ± 0.008	0.007	2.947 ± 0.019	4.815 ± 0.018	0.934	2.953 ± 0.020	4.821 ± 0.019	0.934
3	4.904 ± 0.007	4.912 ± 0.007	0.004	2.811 ± 0.020	4.924 ± 0.019	1.057	2.822 ± 0.021	4.913 ± 0.018	1.046
4	4.884 ± 0.007	4.856 ± 0.007	-0.014	2.637 ± 0.019	4.721 ± 0.020	1.042	2.682 ± 0.020	4.728 ± 0.020	1.023
5	4.895 ± 0.008	4.883 ± 0.008	-0.006	2.783 ± 0.018	4.696 ± 0.019	0.957	2.807 ± 0.018	4.692 ± 0.018	0.943
6	4.894 ± 0.008	4.892 ± 0.008	-0.001	2.751 ± 0.020	4.824 ± 0.020	1.037	2.770 ± 0.020	4.820 ± 0.019	1.025
7	4.882 ± 0.008	4.885 ± 0.008	0.002	3.067 ± 0.018	5.008 ± 0.019	0.971	3.088 ± 0.018	5.019 ± 0.018	0.966
1"	4.866 ± 0.006	4.870 ± 0.008	0.002	3.088 ± 0.017	4.810 ± 0.019	0.861	3.108 ± 0.018	4.813 ± 0.018	0.853
8	4.869 ± 0.007	4.853 ± 0.008	-0.008	2.563 ± 0.021	4.883 ± 0.022	1.160	2.600 ± 0.020	4.879 ± 0.021	1.140

Table S9. Heme-to-heme electron transfer rates for Omc- E, S, and Z surveyed from the literature.[1, 3, 4] Prime and double prime marks are sued to respectively indicate a heme from the preceding or proceeding subunit in the filament.

		Guberman-Pfeffer		Jiang *et al.*		Dahl *et al.*	
Heme Pair	Packing Designation	$k_{\rightarrow}$	$k_{\leftarrow}$	$k_{\rightarrow}$	$k_{\leftarrow}$	$k_{\rightarrow}$	$k_{\leftarrow}$
OmcE							
4'↔1	S	2.5E+07	3.7E+10				
1↔2	T	1.6E+07	3.7E+06				
2↔3	S	4.6E+10	3.3E+10				
3↔4	T	1.9E+08	4.1E+06				
4↔1’’	S	5.2E+07	6.9E+08				
OmcS							
6'↔1	S			5.0E+09	4.0E+07		
1↔2	T	1.2E+06	2.1E+06	2.0E+06	5.0E+07	2.7E+10	1.1E+09
2↔3	S	2.1E+10	1.1E+10	4.0E+09	3.0E+09	9.0E+09	1.8E+10
3↔4	T	1.0E+07	5.2E+09	4.0E+08	2.0E+07	1.3E+07	4.2E+10
4↔5	S	3.4E+09	3.0E+08	1.0E+09	5.0E+09	2.7E+07	3.6E+10
5↔6	T	1.9E+08	1.2E+07	2.0E+06	7.0E+07	7.2E+10	2.9E+03
6↔1"	S	1.5E+09	8.3E+08			9.2E+07	6.7E+09
OmcZ							
7'↔1	S	9.8E+07	1.5E+11				
1↔2	T	5.1E+07	2.5E+09				
2↔3	S	6.7E+07	1.4E+08				
3↔4	S	5.2E+06	2.9E+09				
4↔5	T	3.7E+08	6.8E+07				
5↔6	S	8.1E+08	1.4E+08				
6↔7	T	8.7E+09	4.0E+05				

7↔1’’	S	1.7E+08	5.9E+09

Table S10. Comparison of measured and computed heme-to-heme electron transfer rates in various multi-heme cytochromes[1, 3, 4, 7, 8]

	T-stacked			Slip-stacked		
	Min.	Max.	Avg.	Min.	Max.	Avg.
Experiment						
van Wonderen	8.7E+6	1.3E+8	8.4E+7	3.3E+6	1.1E+10	2.8E+9
OmcE						
Guberman-Pfeffer	3.7E+06	1.9E+08	5.3E+07	2.5E+07	4.6E+10	1.95E+10
OmcS						
Guberman-Pfeffer	1.2E+06	5.2E+09	9.0E+08	3.0E+08	2.1E+10	6.34E+09
Jiang et al.	2.0E+06	4.0E+08	9.1E+07	4.0E+07	5.0E+09	3.01E+09
Dahl et al.	2.9E+03	7.2E+10	2.4E+10	2.7E+07	3.6E+10	1.16E+10
OmcZ						
Guberman-Pfeffer	4.00E+05	8.70E+09	1.95E+09	5.20E+06	1.50E+11	1.60E+10

Table 11. Simulated steady-state, protein-limited electron fluxes in hypothetical chains of all T-stacked, all slip-stacked, alternating T- and slip-stacked (as in OmcS, OmcE, A3MW92, and F2KMU8), or mixed T- and slip-stacked (as in OmcZ) heme groups as a function of the number of heme-to-heme electron transfer steps. The forward and backward rates constants were assumed to be 1×10^8 and 1×10^9 s^{-1} for T- and slip-stacked pairs, respectively. The data in this table were generated with the kinetics model of Blumberger and co-workers[3, 9, 10] and fit to the expected hopping dependence $k_{et} = \frac{A}{N^\eta}$ to generate the right-hand-side of Figure 2 in the main text. In this expression, k_{et} is the overall electron transfer rate through the filament, A is a proportionality constant, N is the number of heme-to-heme electron transfer steps, and η describes the nature of the transport on a scale form 1.0 (diffusive) to 2.0 (directed). The overall k_{et} was converted to a current by multiplying by the charge of an electron (1.602×10^{-19} C/electron).

Step #	All T-stacked	Alternating	Mixed	Slip-stacked
1	1.00×10^8	1.00×10^8	1.00×10^8	9.98×10^8
2	5.00×10^7	9.09×10^7	9.09×10^7	5.00×10^8
3	3.33×10^7	4.76×10^7	8.33×10^7	3.33×10^8
4	2.50×10^7	4.55×10^7	4.55×10^7	2.50×10^8
5	2.00×10^7	3.12×10^7	4.35×10^7	2.00×10^8
6	1.67×10^7	3.03×10^7	3.03×10^7	1.67×10^8
7	1.43×10^7	2.33×10^7	2.94×10^7	1.43×10^8
8	1.25×10^7	2.27×10^7	2.27×10^7	1.25×10^8
9	1.11×10^7	1.85×10^7	2.22×10^7	1.11×10^8
10	1.00×10^7	1.82×10^7	2.17×10^7	1.00×10^8
11	9.09×10^6	1.54×10^7	1.79×10^7	9.09×10^7
12	8.33×10^6	1.52×10^7	1.75×10^7	8.33×10^7
13	7.69×10^6	1.32×10^7	1.49×10^7	7.69×10^7
14	7.14×10^6	1.30×10^7	1.47×10^7	7.14×10^7
15	6.67×10^6	1.15×10^7	1.28×10^7	6.67×10^7
16	6.25×10^6	1.14×10^7	1.27×10^7	6.25×10^7
17	5.88×10^6	1.02×10^7	1.25×10^7	5.88×10^7
18	5.56×10^6	1.01×10^7	1.11×10^7	5.55×10^7
19	5.26×10^6	9.17×10^6	1.10×10^7	5.26×10^7
20	5.00×10^6	9.09×10^6	9.90×10^6	5.00×10^7
21	4.76×10^6	8.33×10^6	9.80×10^6	4.76×10^7
22	4.55×10^6	8.26×10^6	8.93×10^6	4.55×10^7

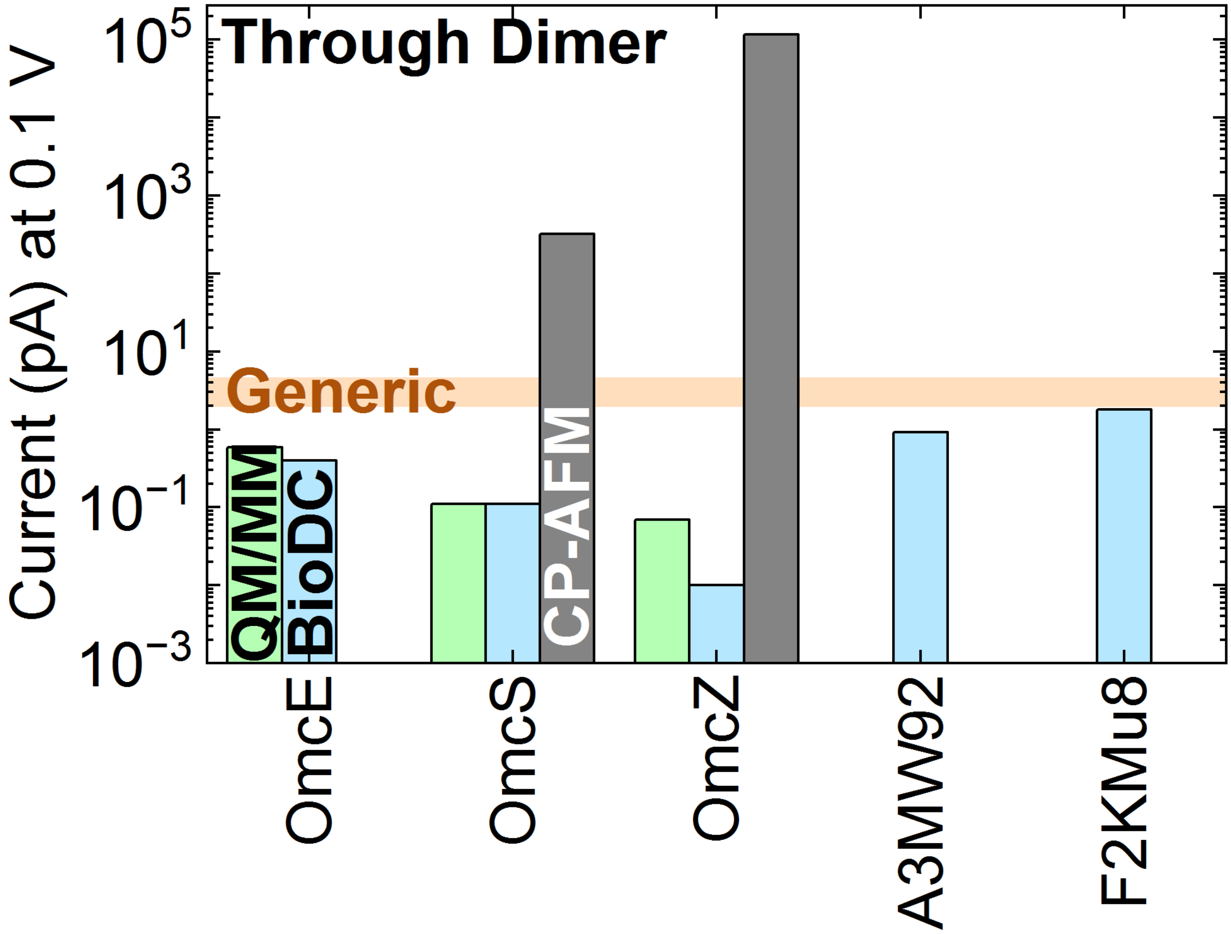

Figure S2. Comparison of simulated protein-limited stead-state electrical currents through a generic chain of alternating T- and slip-stacked hemes and the known cytochrome filaments to the currents that would be observed for filaments of the same length based on the experimentally reported conductivities. The heme chains are taken to have a main cofactor chain of 8 – 14 hemes. The quantum mechanical/molecular mechanical (QM/MM) and BioDC computational results on which this figure is based were published in Refs. 1 and [11]. The conducting-probe atomic force microscopy (CP-AFM) reported conductivities come from Refs. 4, [12], and [13].

Derivation of Diffusion Constants from Experimentally Measured Conductivities

An expression that relates the diffusion constant (D) to the experimental conductance (G) is Eq. S1.

$$\mathrm{D} = \frac{\mathrm{Gk_bTL_w}}{\mathrm{A\rho e^2}} \tag{S1}$$

where $\mathrm{k_b}$, e, and T are respectively the Boltzmann constant, the elementary charge, and temperature, respectively; A and $\mathrm{L_w}$ are the cross-sectional area and length of the conduction channel; ρ is the charge density; and G is the conductance.

Assuming the conduction channel through the helical filament is cylindrical and that the charges are only transported through the heme chain, $\mathrm{A} = \pi \mathrm{r}^2$. where $\mathrm{r} = 0.75\ \mathrm{nm}$ for the heme group. $\mathrm{L_w}$ is taken as 300 nm, which is a typical electrode spacing in experiments. The ρ that is associated with maximal charge flux is $\frac{\mathrm{N_{heme}}}{2}\frac{1}{\mathrm{AL_{sub}}}$, where $\mathrm{N_{heme}}$ is the number of hemes in a subunit of the homopolymer and $\mathrm{L_{sub}}$ is the length of a subunit. From the cryogenic electron microscopy-resolved structures, $\mathrm{L_{sub}}$ is 4.67 nm for OmcS and 5.8 nm for OmcZ. Conducting probe atomic force microscopy experiments report G to be 1.10×10^{-10} S for OmcS and 4.91×10^{-8} S for OmcZ.

Evaluating Eq. S1 with the foregoing values gives electron diffusion constants of 8.3 $\mathrm{nm^2/ns}$ for OmcS and 3699 $\mathrm{nm^2/ns}$.

Derivation of a Diffusion Constant from the Cellular Respiratory Rate

A *Geobacter sulfurreducens* cell can metabolize $\sim 6 \times 10^5$ (acetate molecules)/s. Oxidation of each acetate molecule yields 8 electrons, 95% of which is discharged by the cell. It follows that the cell discharges $0.95(6 \times 10^5 \times 8) \approx 5 \times 10^6$ electrons/s, or a current of $\mathrm{I} \approx (5 \times 10^6\ \mathrm{electrons/s})(1.602 \times 10^{-19}\ \mathrm{C/s})$ or 0.73 pA. When the cell discharges this current, there is roughly a 0.1 V difference between the intra- and extra-cellular reduction-oxidation (redox) half reactions. Thus, the needed conductance of the electrical conduit used by the cell is $\mathrm{G} = \frac{7.3 \times 10^{-13}\ \mathrm{A}}{0.1\ \mathrm{V}}$ or 7.3×10^{-12} S.

Evaluating Eq. S1 with this conductance and all the other variables as defined above gives a diffusion constant of 0.6 nm^2/ns.

Derivation of Diffusion Constants from Experimental Electron Transfer Rates between Hemes in Highly Conserved Packing Geometries

kinetic analyses of ultrafast transient absorption measurements on photosensitized variants of the small tetraheme cytochrome (STC)[68] and the metal reducing cytochrome type C (MtrC)[69] from *Shewanella oneidensis* indicate that the electron transfer rates within T- or slip-stacked heme pairs are respectively 8.7×10^{6} – 1.3×10^{8} $(\text{Avg.}\, 8.4 \times 10^{7})$ s^{-1} and 3.3×10^{6} – 1.1×10^{10} $(\text{Avg.}\, 2.8 \times 10^{9})$ s^{-1}. Roughly speaking, then, the rates tend in fall in the 1.0 – 10.0 ns range. Because all known cytochrome filaments have very nearly an equal proportion of T- and slip-stacked heme pairs, it seems reasonable to assume that the average rate through the filament is $\frac{(1\times10^{8}+1\times10^{9})}{2} = 6 \times 10^{8}$ s^{-1}. If each electron transfer is assumed to proceed at this averaged rate between hemes with an average edge-to-edge spacing of 0.5 nm, as in the cytochrome filaments, the corresponding diffusion constant is $\text{D} = (6 \times 10^{8}\ \text{s}^{-1})(0.5\ \text{nm})^{2}$ or 0.1 nm^2/ns. This result is a good order-of-magnitude estimate, coming within a factor of 6 of the diffusion constant required by cellular respiration.

Derivation of the Electron Diffusion Constant from Prior Electron Transfer Calculations

The average computed electron transfer rates within T-stacked or slip-stacked heme pairs in the outer membrane cytochrome (Omc) types E, S, and Z filaments fall in the range 5×10^7 – 2×10^9 s^{-1} and 6×10^9 – 2×10^{10} s^{-1}. More specifically, the average of the rates for the two packing geometries gives diffusion constants of 0.9 (OmcS), 2.2 (OmcZ), and 2.4 (OmcE) nm^2/ns. All of these diffusion constants are somewhat greater than the 0.6 nm^2/ns needed for cellular respiration, indicating that the flow of electrons through the filaments is not rate-limiting for the organism.

References

(1) Guberman-Pfeffer, M. J. Structural Determinants of Redox Conduction Favor Robustness over Tunability in Microbial Cytochrome Nanowires. *J. Phys. Chem. B* **2023**, *127* (32), 7148-7161. DOI: 10.1021/acs.jpcb.3c02912 From NLM Medline.

(2) Baquero, D. P.; Cvirkaite-Krupovic, V.; Hu, S. S.; Fields, J. L.; Liu, X.; Rensing, C.; Egelman, E. H.; Krupovic, M.; Wang, F. Extracellular cytochrome nanowires appear to be ubiquitous in prokaryotes. *Cell*, 2023; Vol. 186, pp 1-12.

(3) Jiang, X.; van Wonderen, J. H.; Butt, J. N.; Edwards, M. J.; Clarke, T. A.; Blumberger, J. Which Multi-Heme Protein Complex Transfers Electrons More Efficiently? Comparing MtrCAB from Shewanella with OmcS from Geobacter. *J. Phys. Chem. Lett.* **2020**, *11* (21), 9421-9425. DOI: 10.1021/acs.jpclett.0c02842.

(4) Dahl, P. J.; Yi, S. M.; Gu, Y.; Acharya, A.; Shipps, C.; Neu, J.; O'Brien, J. P.; Morzan, U. N.; Chaudhuri, S.; Guberman-Pfeffer, M. J. A 300-fold conductivity increase in microbial cytochrome nanowires due to temperature-induced restructuring of hydrogen bonding networks. *Sci. Adv.* **2022**, *8* (19), eabm7193.

(5) OBrien, J. P. A Tale of Two Nanowires: the Biochemical and Spectroscopic Characterization of the Conductive Cytochrome OmcS and OmcZ Filaments of Geobacter Sulfurreducens. Yale University, 2020.

(6) Guberman-Pfeffer, M. J. Assessing Thermal Response of Redox Conduction for Anti-Arrhenius Kinetics in a Microbial Cytochrome Nanowire. *J. Phys. Chem. B* **2022**, *126* (48), 10083-10097. DOI: 10.1021/acs.jpcb.2c06822.

(7) van Wonderen, J. H.; Hall, C. R.; Jiang, X.; Adamczyk, K.; Carof, A.; Heisler, I.; Piper, S. E. H.; Clarke, T. A.; Watmough, N. J.; Sazanovich, I. V.; et al. Ultrafast Light-Driven Electron Transfer

in a Ru(II)tris(bipyridine)-Labeled Multiheme Cytochrome. *J. Am. Chem. Soc.* **2019**, *141* (38), 15190-15200. DOI: 10.1021/jacs.9b06858 From NLM Medline.

(8) van Wonderen, J. H.; Adamczyk, K.; Wu, X.; Jiang, X.; Piper, S. E. H.; Hall, C. R.; Edwards, M. J.; Clarke, T. A.; Zhang, H.; Jeuken, L. J. C.; et al. Nanosecond heme-to-heme electron transfer rates in a multiheme cytochrome nanowire reported by a spectrally unique His/Met-ligated heme. *Proc. Natl. Acad. Sci. U.S.A.* **2021**, *118* (39). DOI: 10.1073/pnas.2107939118.

(9) Breuer, M.; Rosso, K. M.; Blumberger, J. Electron flow in multiheme bacterial cytochromes is a balancing act between heme electronic interaction and redox potentials. *Proc. Natl. Acad. Sci. U.S.A.* **2014**, *111* (2), 611-616. DOI: 10.1073/pnas.1316156111.

(10) Jiang, X.; Futera, Z.; Ali, M. E.; Gajdos, F.; von Rudorff, G. F.; Carof, A.; Breuer, M.; Blumberger, J. Cysteine Linkages Accelerate Electron Flow through Tetra-Heme Protein STC. *J. Am. Chem. Soc.* **2017**, *139* (48), 17237-17240. DOI: 10.1021/jacs.7b08831.

(11) Guberman-Pfeffer, M. J. From Hot Water to Dry Dirt: Microbes Use Cytochrome'Nanowires' of Similar Conductivity but Different Structure. *bioRxiv.* **2023**, 2023.2006. 2012.544705.

(12) Wang, F.; Gu, Y.; O'Brien, J. P.; Sophia, M. Y.; Yalcin, S. E.; Srikanth, V.; Shen, C.; Vu, D.; Ing, N. L.; Hochbaum, A. I. Structure of microbial nanowires reveals stacked hemes that transport electrons over micrometers. *Cell.* **2019**, *177* (2), 361-369. e310.

(13) Yalcin, S. E.; O'Brien, J. P.; Gu, Y.; Reiss, K.; Yi, S. M.; Jain, R.; Srikanth, V.; Dahl, P. J.; Huynh, W.; Vu, D.; et al. Electric field stimulates production of highly conductive microbial OmcZ nanowires. *Nat. Chem. Biol.* **2020**, *16* (10), 1136-1142. DOI: 10.1038/s41589-020-0623-9.
